# Supplementary material for: Resolving the full spectrum of human genome variation using Linked-Reads
Source: Genome Res. 2019 Apr;29(4):635–45. doi: 10.1101/gr.234443.118 (PMC6442396; doi:10.1101/gr.234443.118)
Supplement: Supplemental Material [file supp_gr.234443.118_Supplemental_longranger-2.2.zip › longranger-2.2.tar.bz2!/lr2.2-public/lib/stan/cmdstan/stan/doc/api/html/matrix_2max_8hpp_source.html]

Stan: src/stan/math/matrix/max.hpp Source File


|  |  |
| --- | --- |
|  | Stan  2.5.0  probability, sampling & optimization |


- Main Page
- Related Pages
- Namespaces
- Classes
- Files

- File List
- File Members

All Classes Namespaces Files Functions Variables Typedefs Enumerations Enumerator Friends Macros Pages

- src
- stan
- math
- matrix

max.hpp

Go to the documentation of this file.

1 #ifndef STAN\_\_MATH\_\_MATRIX\_\_MAX\_HPP

2 #define STAN\_\_MATH\_\_MATRIX\_\_MAX\_HPP

3

4 #include <stdexcept>

5 #include <vector>

6 #include <stan/math/matrix/Eigen.hpp>

7

8 namespace stan {

9  namespace math {

10

19  inline int max(const std::vector<int>& x) {

20  if (x.size() == 0)

21  throw std::domain\_error("error: cannot take max of empty int vector");

22  int max = x[0];

23  for (size\_t i = 1; i < x.size(); ++i)

24  if (x[i] > max)

25  max = x[i];

26  return max;

27  }

28

36  template <typename T>

37  inline T max(const std::vector<T>& x) {

38  if (x.size() == 0)

39  return -std::numeric\_limits<T>::infinity();

40  T max = x[0];

41  for (size\_t i = 1; i < x.size(); ++i)

42  if (x[i] > max)

43  max = x[i];

44  return max;

45  }

46

53  template <typename T, int R, int C>

54  inline T max(const Eigen::Matrix<T,R,C>& m) {

55  if (m.size() == 0)

56  return -std::numeric\_limits<double>::infinity();

57  return m.maxCoeff();

58  }

59

60  }

61 }

62 #endif

stan::math::max

double max(const double a, const double b)

**Definition:** max.hpp:7

Eigen.hpp


---

[ Stan Home Page ]

*© 2011–2014,
Stan Development Team.*
